# Cerebrovascular disease emerges with age and Alzheimer’s disease in adults with Down syndrome

**DOI:** 10.1038/s41598-024-61962-y

**Published:** 2024-05-29

**Authors:** Patrick Lao, Natalie Edwards, Lisi Flores-Aguilar, Mohamad Alshikho, Batool Rizvi, Dana Tudorascu, H. Diana Rosas, Michael Yassa, Bradley T. Christian, Mark Mapstone, Benjamin Handen, Molly E. Zimmerman, Jose Gutierrez, Donna Wilcock, Elizabeth Head, Adam M. Brickman

**Affiliations:** 1 Gertrude H. Sergievsky Center and Taub Institute for Research on Alzheimer’s Disease and the Aging Brain, College of Physicians and Surgeons, Columbia University, 630 West 168th Street, PS Box 16, New York, NY 10032 USA; 2 Department of Neurology, College of Physicians and Surgeons, Columbia University, New York, NY 10032 USA; 3 Department of Pathology and Laboratory Medicine, University of California, Irvine, Irvine, CA 92697 USA; 4 Department of Neurology, University of California, Irvine, Irvine, CA 92697 USA; 5 Department of Psychiatry, University of Pittsburgh Medical Center, Pittsburgh, PA 15213 USA; 6 Department of Neurology, Massachusetts General Hospital, Harvard Medical Center, Boston, MA 02114 USA; 7 Waisman Center, University of Wisconsin-Madison, Madison, WI 53705 USA; 8 Department of Psychology, Fordham University, Bronx, NY 10458 USA; 9 Departments of Neurology and Anatomy, Cell Biology, and Physiology, Stark Neurosciences Research Institute, Indiana University School of Medicine, Indianapolis, IN USA; 10 Department of Neurobiology and Behavior, University of California, Irvine, Irvine, CA 92697 USA

**Keywords:** Biomarkers, Diseases of the nervous system

## Abstract

Adults with Down syndrome have a genetic form of Alzheimer’s disease (AD) and evidence of cerebrovascular disease across the AD continuum, despite few systemic vascular risk factors. The onset and progression of AD in Down syndrome is highly age-dependent, but it is unknown at what age cerebrovascular disease emerges and what factors influence its severity. In the Alzheimer’s Biomarker Consortium-Down Syndrome study (ABC-DS; n = 242; age = 25–72), we estimated the age inflection point at which MRI-based white matter hyperintensities (WMH), enlarged perivascular spaces (PVS), microbleeds, and infarcts emerge in relation to demographic data, risk factors, amyloid and tau, and AD diagnosis. Enlarged PVS and infarcts appear to develop in the early 30s, while microbleeds, WMH, amyloid, and tau emerge in the mid to late 30s. Age-residualized WMH were higher in women, in individuals with dementia, and with lower body mass index. Participants with hypertension and APOE-ε4 had higher age-residualized PVS and microbleeds, respectively. Lifespan trajectories demonstrate a dramatic cerebrovascular profile in adults with Down syndrome that appears to evolve developmentally in parallel with AD pathophysiology approximately two decades prior to dementia symptoms.

## Introduction

Although typically considered a disorder that affects early life intellectual and physical development, Down syndrome is also associated with increased risk for Alzheimer’s disease (AD) in later life. The link between Down syndrome and AD was first uncovered in the 1940s^1^ and subsequently attributed to the triplication of the 21st chromosome^2^, which contains the amyloid precursor protein gene (*APP*). With chromosome 21 trisomy comes increased production and aggregation of beta-amyloid protein, one of the primary pathological features of AD^3–6^. Indeed, individuals with Down syndrome overproduce beta-amyloid protein from birth^7^ and by the time they are in their 40 s, most have the full pathological features of AD. Today, Down syndrome is considered a “genetic form” of AD, together with fully penetrant, autosomal dominant familial genetic mutations^8–14^.

Understanding the emergence and progression of AD-related features across the adult lifespan in Down syndrome is critical for two reasons. First, significant recent advances in medical care for individuals with Down syndrome have resulted in an average lifespan that has nearly doubled since the 1980s^15^. As a result, people with Down syndrome are typically living into their 60 s and almost all will suffer from AD dementia within their lifetimes^15^. Thus, AD represents an emerging public health crisis in this population and the time course, risk and pathogenic factors, and potential prevention or treatment targets need to be identified to mitigate its impact. Second, because the biological and clinical progression of AD among individuals with Down syndrome are very similar to late onset AD, the study of AD in this genetically at-risk population has great potential to provide insight into pathogenesis, course, and prevention or treatment strategies for the neurotypical population as well. With advances in care of medical conditions, improved social integration, and a large segment of the Down syndrome population entering older adulthood, AD threatens the health economics and quality of life of an aging society.

There is significant debate about the causes of AD. The field has embraced a single pathogenic pathway, in which accumulation of amyloid leads to tau pathology, subsequent neurodegeneration, and associated cognitive and functional decline. This “amyloid cascade hypothesis” has informed both diagnostic frameworks^12–14^ and primary treatment strategies for AD^16^. However, emerging evidence suggests that the clinical course and, possibly, the pathogenesis of AD are multiply determined^17^. Notably, the vast majority of people who die with symptomatic AD have evidence of significant cerebrovascular disease^18,19^. Cerebrovascular disease contributes to risk, onset, and clinical course of AD^20,21^ and recent studies show elevated severity of cerebrovascular disease among individuals with autosomal dominant forms of AD^22,23^. Supported by animal experiments that suggest that cerebrovascular disease gives rise to AD pathological features^24^ and genetic studies linking vascular factors to AD prevalence^25^, emerging evidence suggests that cerebrovascular disease is a core feature of AD.

Despite having low rates of systemic vascular risk factors, such as hypertension, we previously showed that individuals with Down syndrome have increased magnetic resonance imaging (MRI) markers of cerebrovascular disease, including white matter hyperintensities (WMH), enlarged perivascular spaces (PVS), cerebral microbleeds, and infarcts, which increase as a function of clinical AD diagnosis and its antecedent clinical conditions^26^ and may be mediated in part by inflammatory processes that result in neurodegeneration^27,28^. Notably, even in individuals without clinical symptoms of AD, we observed a significant degree of cerebrovascular changes, suggesting that they emerged earlier in life and may precede or coincide with the emergence of classical AD pathophysiology. Consistent with work in late onset and autosomal dominant AD, these findings highlight the centrality of cerebrovascular disease to the presentation of AD and possibly to its pathogenesis.

Like other genetically deterministic forms of AD, the development of AD pathological features and subsequent symptoms in Down syndrome is age-dependent and follows a somewhat prescribed pattern, highlighting that AD is a developmental component of Down syndrome. Despite our initial observations of an association between cerebrovascular disease and AD diagnosis^26^, it is unclear at what age cerebrovascular features typically arise in adults with Down syndrome and how the temporal evolution of cerebrovascular disease compares with other AD biomarkers, like amyloid and tau. In the current study, we examined the age “inflection point” at which MRI-based cerebrovascular disease markers emerge in adults with Down syndrome, characterizing them relative to the temporality of amyloid and tau biomarkers, measured with positron emission tomography (PET). We also examined vascular and AD-related factors that could explain a greater-than-expected cerebrovascular biomarker for a given age.

## Results

**Figure 1:**
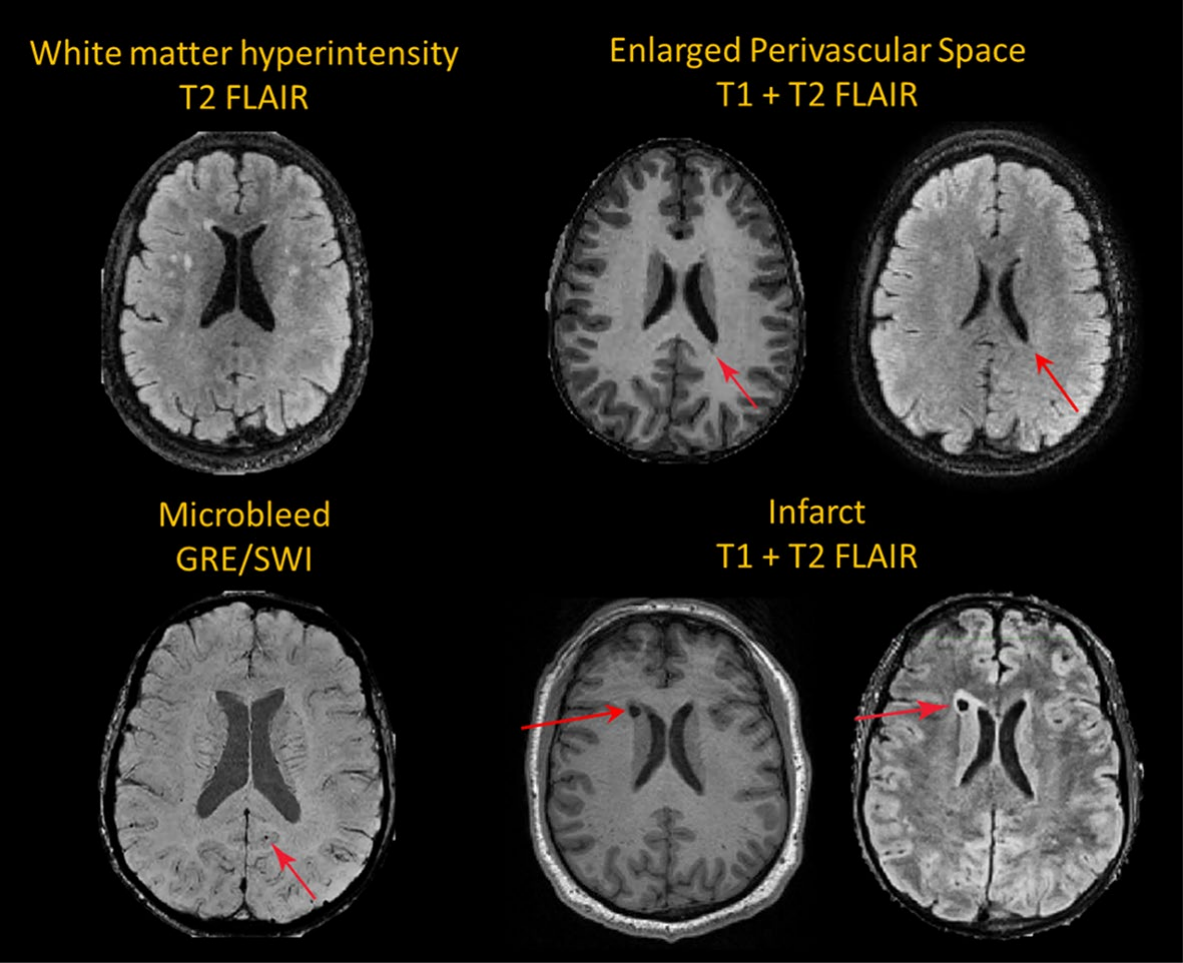
Representative MRI scans for white matter hyperintensity volume, enlarged perivascular spaces, microbleeds, and infarcts across the lifespan of adults with Down syndrome.

Older age (F(2,239) = 40.3, *p* < 0.001), greater amyloid burden (F(2,170) = 82.3, *p* < 0.001), and greater tau burden (Braak I/II: F(2,141) = 47.5, *p* < 0.001; Braak III/IV: F(2,141) = 55.9, *p*-value < 0.001; Braak V/VI: F(2,141) = 27.2, *p* < 0.001) were associated with more advanced AD-related diagnostic group from Cognitively-Stable to MCI-DS and AD dementia, highlighting the strong age-dependency of disease-related factors in this population (Table 1). The proportion of participants with hyperlipidemia was higher in those with AD dementia compared with those characterized as Cognitively-Stable; BMI was lower in those with MCI-DS compared with those characterized as cognitively-stable; and obstructive sleep apnea was common in all diagnostic groups (Table 1). Despite low frequencies of traditional vascular risk factors including hypertension (6.2%) and diabetes (5.4%), WMH volume (F(2,239) = 9.8, *p* < 0.001), enlarged PVS scores (F(2,167) = 5.4, *p* = 0.005), and the presence of infarcts (χ^2^(2) = 7.2, *p* = 0.03), but not the presence of microbleeds (﻿χ^2^(2) = 4.9, *p* = 0.09) were associated with more advanced diagnostic group, as reported previously in a subset of the older participants^26^. Figure 1 displays representative images from each MRI modality.

**Table 1:** Alzheimer’s disease progression markers, demographic variables, and vascular risk factors in adults with Down syndrome in the whole sample and by consensus diagnosis groups.

	Total	Cognitively-stable	MCI-DS	AD Dementia	Statistics
N	242(100%)	182 (7S.2%)	30 (12.4%)	30(12.4%)	
Age‡	25–72	25–72	43–63	44–68	**F[2,239) = 40.3,** ***p*-value < 0.001**
	45.1 [43.9,46.4]	42.4 [41.1, 43.7]	51.3 [49.2, 53.4]	55.5 [53.3, 57.7]	
Centiloid‡	− 19.2 to 194.2	− 19.2 to 145.5	15.3–162.1	50.5–194.2	**F(2,170) = 82.3,** ***p*-value = le−25**
	34.3 [29.0, 39.5]	19.0115.0, 23.1]	76.4 [62.2, 90.5]	99.2 186.7, 111.6]	
Early Braak‡	0.9–2	0.9–1.7	1–1.9	1.2–2	**F(2,141) = 47.S,** ***p*-value = 2e**−16
	1.3 [1.23,1.29]	1,2 [1.17, 1.22]	1.6 [1.46,1.65]	1.6 [1,54, 1,75]	
Middle Braak‡	0.9–2.2	0.9–1.7	1–2.2	1–2.2	**F(2,141) = 55.9,** ***p*-value = le−18**
	1.2 [1.16, 1.23]	1.1 [1.11, 1.14]	1.5 [1.35, 1.61]	1.6 11.51, 1.79]	
Late Braak‡	0.5–2.5	0.9–1.5	1–2.2	0.5–2.5	**F(2,141) = 27.2,** ***p*-value=le−10**
	1.1 [1,06, 1.13]	1 [1,02, 1,06]	1.3 **[**1.18, 1.44|	**1**.4 [1.25, 1,64]	
Sex	103 (42.6%) women	84 (46.2%) women	7 (23.3%) women	12 (40%) women	﻿χ^2^(2) = 5.6, *p*-value = 0.06
	139(57.4%) men	98(53.8%) men	23 (76.7%) men	18 (60%) men	
Ethnicity	231 (95,5%) Non-Hispanic	171 (94%) Non-Hispanic	30 (94%) Non-Hispanic	30 (94%) Non-Hispanic	﻿χ^2^ = 3.8, *p*-value = 0.15
	11(4.5%) Hispanic	11(6%) Hispanic	0 (0%) Hispanic	0 (0%) Hispanic	
Race	231(97.1%) White 4(1.7%) Black 3 (1,3%) Asian	175 (98.3%) White 1(0.6%) Black 2(1.1%) Asian	28 (93.3%) White 1 (3.3%) Black 1(3.3%) Asian	28 (93.3%) White 2 (3.3%) Black 0 (0%) Asian	﻿χ^2^(4) = 7.8, *p*-value = 0.1
Premorbid intellectual developmental disability	129(53.5%) Mild 95 (39.4%) Moderate 17 (7.1%) Severe	97(53.6%) Mild 70 (38.7%) Moderate 14 (7.7%) Severe	15 (50%) Mild 14(46.7%) Moderate 1 (3.3%) Severe	17 (56.7%) Mild 11(36.7%) Moderate 2 (6.7%) Severe	﻿χ^2^(2) = 1.3, *p*-value = 0.86
APOE4	55 (24.8%) Carrier	37 (22.3%) Carrier	6(23.1%) Carrier	12 (40%) Carrier	﻿χ^2^(2) = 4.3, *p*-value = 0.12
Hypertension	12 (6.2%) Yes	6 (4%) Yes	3 (12%) Yes	3 (15.8%) Yes	﻿χ^2^(2) = 5.6, *p*-value = 0.06
Diabetes	13 (5.4%) Yes	9 (4.9%) Yes	2 (6.9%) Yes	2 (6.7%) Yes	﻿χ^2^(2) = 0.3, *p*-value = 0.86
Hyperlipidemia†	SI (42%) Yes	55 (36.9%) Yes	13 (52%) Yes	13 (68.4%) Yes	**﻿χ**^**2**^(2) **= 8.1**, ***p*-value = 0.02**
BMI^+^	18.3–69.2	18.3–69.2	20.8–39.4	20.9–42.2	**F(2,231) = 3.9,** ***p*-value = 0,02**
	31.9 [30.9, 32.9]	32,7 [31.5, 33.9]	28,9 [27.3, 30.5]	30.3 [28.2, 32.4]	
Obstructive Sleep Apnea	95 (43.6%) Yes	69 (41.8%) Yes	10(37%)Yes	16 (61.5%) Yes	﻿χ2(2) = 4.1, *p*-value = 0.13

Bold indicates significance in the omnibus test. ^+^indicates significance between Cognitively-Stable and MCI-DS. † indicates significance between Cognitively-Stable and AD. ‡ indicates significance between Cognitively-Stable and MCI-DS and between Cognitively-Stable an AD.

**Figure 2:**
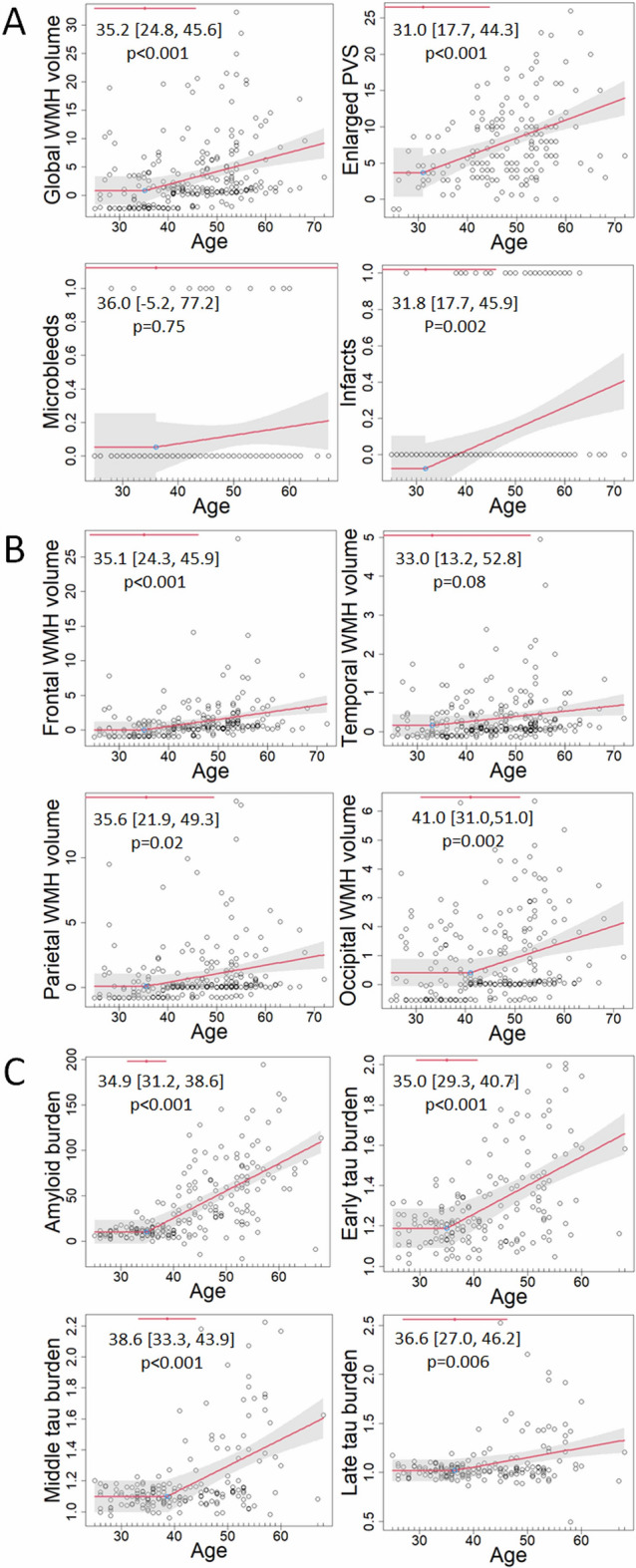
Piecewise left-null regressions of (**A**) cerebrovascular biomarkers, (**B**) regional white matter hyperintensity volume, and (**C**) traditional AD biomarkers against age across the lifespan in adults with Down syndrome. Inflection point estimates, their 95% confidence interval, and their *p*-value are displayed at the top.

Piecewise left-null regression models were fit to each cerebrovascular biomarker across the lifespan to test the hypothesis that each cerebrovascular disease biomarker emerges at a given age, reflecting disease progression in adults with DS (Fig. 2). Enlarged PVS and the presence of infarcts were the biomarkers showing the earliest age-associated increase at 31 and 32 years old, respectively. Global WMH inflected at 35 years old. Regionally, frontal and parietal WMH inflected at 35 years old, while occipital WMH inflected later at 41 years old. In relation to traditional AD biomarkers, global amyloid and tau burden in early Braak regions, measured with molecular positron emission tomography (PET) imaging, inflected at 35 years old, after enlarged PVS scores. Then, tau burden in middle and late Braak regions inflected at 39 and 37 years old, respectively, after WMH.

We examined the association of age-residualized cerebrovascular biomarker levels, which reflect the extent to which the biomarker severity deviates from the value predicted by age, with demographic variables, vascular risk factors, amyloid and tau burden, and AD diagnosis (Table 2). Women had larger residuals compared with men for global WMH, driven by parietal, temporal, and occipital WMH. APOE-ε4 carriers had larger residuals compared with non-carriers for the presence of microbleeds. Individuals with hypertension had larger residuals compared with those without hypertension for enlarged PVS scores. Greater BMI was associated with lower age-residualized global WMH, driven by temporal WMH. Individuals with AD dementia had larger residuals compared with those who were cognitively-stable for global WMH, driven by parietal, temporal, and occipital WMH.

**Table 2:** Associations of demographic variables, vascular risk factors, amyloid and tau burden, and AD diagnosis with age-residualized cerebrovascular disease biomarkers from piecewise left-null models.

	Global WMH	Frontal WMH	Temporal WMH	Parietal WMH	Occipital WMH	PVS	MB	Infarct
Sex **(F)**	**2.13 [0.72,3.55|**	0,61 [− 0.06,1.28]	**0.19 [0.04,0.34]**	**0.88 [0.32,1.43]**	**0.4 [0.06,0.73]**	− 0.84 [− 2.3,0.61]	− 9E−03 [− 0.12,0.1]	0,07 [− 0.02,0.15]
	***p*** = **3E**−**03**	*p* = 0.07	***p*** = **0.01**	***p*** = **2E**−**03**	***p*** = **0.02**	*p*** = **0.26	*p*** = **0.87	*p*** = **0.14
AP0E(ε4)	0.52 [− 1.26,2.3] *p*** = **0.57	0.22 [− 0.61.1.05] *p*** = **0.6	0.07 [− 0.12,0.26] *p*** = **0.48	0.04 [− 0.66,0.74] *p*** = **0.91	0.17 [− 0.25,0.59] *p*** = **0.43	0.28 [− 1.44,2] *p*** = **0.75	**0.13 [2E**−**03,0.26] *p*** = **0.05**	− 0.04 [− 0.15,0.06] *p*** = **0.43
Hypertension	− 1.02 [− 4 17,2 13]	− 0.87 [− 2.04,0 29]	− 0.08 [− 0.44,0.27]	0.04 [− 1.26,134]	− 0.07 [− 0.87,0 73]	**3.49 [0.15,6.83]**	− 0.16 [− 0.43,0.11]	− 0.1 [− 0.3,0.1]
	*p*** = **0.53	*p*** = **0.14	*p*** = **0.64	*p*** = **0.95	*p*** = **0.87	***p*** = **0.04**	*p*** = **0.25	*p*** = **0.33
Diabetes	− 1.01 [− 4.17,2.16]	− 0.66 [− 2.13,0.82]	− 0.03 [− 0.37,0.3]	− 0.35 [− 1.59,0.89]	0.02 [− 0.74,0.77]	− 2.03 [− 5.32,1.26]	0.06 [− 0.21,0.33]	− 0.13 [− 0.33,0.07]
	*p*** = **0.53	*p*** = **0.39	*p*** = **0.85	*p*** = **0.58	*p*** = **0.97	*p*** = **0.23	*p*** = **0.66	*p*** = **0.19
Hyperlipidemia	− 0,65 [− 2.19,0.89]	− 0.35 [− 0.92,0.22]	− 0.03 [− 0.2,0.15]	− 0.11 [− 0.74,0.53]	− 0.15 [− 0.55,0.24]	− 0.72[− 2.3,0.87]	− 0.07[− 0.2,0.06]	− lE − 03 [− 0.1,0.09]
	*p*** = **0.41	*p*** = **0,24	*p*** = **0.76	*p*** = **0.74	*p*** = **0.44	*p*** = **0,38	*p*** = **0.2B	*p*** = **0,9S
BMI	− **0.1[**− **0.19,** − **6E**−**03]**	− 0.03 [− 0.08,0.01]	− **0.01 [**− **0.02,** − **9E**−**04]**	− 0.03 [− 0.07,56 − 03]	− 0.02[|− 0.04,lE−03]	− 0.1 [− 0.21,0.01]	− 5E − 03 [− 0.01,36 − 03]	8E − 04 [− 5E−03,6E − 03]
	***p*** = **0.04**	*p*** = **0.17	***p*** = **0.03**	*p*** = **0.09	*p*** = **0.07	*p*** = **0.08	*p*** = **0.23	*p*** = **0.78
Obstructive sleep apnea	− 0.39[− 1.86,1.09]	0.28 [− 0.41,0.98]	− 0.09 [− 0.25,0.06]	− 0.28 [− 0.85,0.3]	− 0.26 [− 0.61,0.09]	− 0.25[− 1.8,1.31]	0.11 [− 5E − 03,0.23]	− 0.03 [− 0.12,0.07]
	*p*** = **0.61	*p*** = **0,42	*p*** = **0.24	*p*** = **0.35	*p*** = **0.15	*p*** = **0.76	*p*** = **0.06	*p*** = **0.6
Amyloid	− 2E−03 [− 002,0.02] *p*** = **0.85	− 5E − 03 [− 0.01,4E − O3] *p*** = **0,27	6E − 04 [− 2E−03,3E−03] *p*** = **0.59	7E − 04[− 8E−03,9E−03] *p*** = **0.B8	2E−03 [− 3E−03,7E−03] *p*** = **0.46	0.01 [− 0.01,0.03] *p*** = **0.34	− 4E − 04[− 2E−03,lE − 03] *p*** = **0.6	− 3E − 04[− 2E−03,9E−04] *p*** = **0.61
Braakl/ll	0.21 [− 3.01,3.43]	0.21 [− 1.3,1.71]	− 0.05 [− 0.35,0.25]	− 0.51 [− 1.51,0.48]	0.611 − 0.32,1.54]	− 0.27[− 3.97,3.44]	− 0.11 [− 0.4,0.18]	− 0.02 [− 0.27,0.22]
	*p*** = **0.9	*p*** = **0.79	*p*** = **0.73	*p*** = **0.31	*p*** = **0.2	*p*** = **0.89	*p*** = **0.46	*p*** = **0.84
Braak lll/IV	− 0.09 [− 3.17,3]	0.13 [− 1.31,1.58]	− 0.04 [− 0.33.0.2S]	− 0.49 [− 1.44,0.47]	0.36[− 0.54,1.2S]	1.62 [− 1.95,5,19]	− 0.09 [− 0.36,0.18]	− 0.09 [− 0.33,0.15]
	*p*** = **0.96	*p*** = **0,86	*p*** = **0,78	*p*** = **0.32	*p*** = **0.43	*p*** = **0,38	*p*** = **0.52	*p*** = **0,47
Braak V/VI	0.07 [− 3.12,3.25]	− 0.1 [− 1.59,1.39]	3E − 03 [− 0.29,0.3]	− 0.31[− 1.29,0.68]	0.551 − 0.37,1.47]	1.45 [− 2.23,5.14]	− 0.04[− 0.33,0.25]	− 0.19 [− 0.43,0.05]
	*p*** = **0.97	*p*** = **0.89	*p*** = **0.9S	*p*** = **0.54	*p*** = **0.24	*p*** = **0.44	*p*** = **0.77	*p*** = **0.12
Consensus diagnosis [MCI-DS, AD dementia)	0.51 [− 1.65,2.66]	− 0.2 [− 1.22,0.821	0.17 [− 0.06,0.40]	0.16 [− 0.68,1.00]	0.391 − 0.13,0.90]	− 0.43 [− 2.51,1.65]	− 0.13 [− 0.30,0.04]	− 0.08 [− 0.21,0.05]
	*p*** = **0.65	*p*** = **0.70	*p*** = **0.14	*p*** = **0.71	*p*** = **0.14	*p*** = **0.69	*p*** = **0.13	*p*** = **0.24
	**3.13 [0.98,5.28] *p* = 5E**−**3**	0.85 [− 0.16.1.87] *p*** = **0.10	**0.24 [0.01,0.47] *p*** = **0.04**	**1.45 [0.61, 2.29] *p* = 8E**−**3**	**0.57 [0.06,1.09] *p*** = **0.03**	0.79 [− 1.17,2.74] *p*** = **0.43	0.08 [− 0.07,0.24] *p*** = **0.28	0.09 [− 0.05.0.22] *p*** = **0.21

Bold indicates significance.

## Discussion

We found that markers of cerebrovascular disease emerge in adults with Down syndrome within the same timeframe as amyloid and tau pathology and prior to the onset of AD clinical symptoms. The temporal ordering of inflection points suggests that enlarged PVS and infarcts develop in the early 30 s, while microbleeds, WMH, amyloid, and tau develop in the mid to late 30 s. Several demographic factors, vascular risk factors, and AD diagnosis were associated with a greater amount of cerebrovascular biomarkers for a given age. Therefore, vascular risk factors may exacerbate the extent of cerebrovascular disease, but they are not necessary for cerebrovascular disease to emerge across the lifespan in adults with Down syndrome.

Potential pathways between specific markers of cerebrovascular disease and AD have been studied in adults without Down syndrome^29^ and animal models^30^. Enlarged PVS generally capture impaired clearance glymphatic mechanisms due to chronic exposure to toxins like soluble beta-amyloid and may contribute to further amyloid deposition in the parenchyma^31^. Microbleeds among people with Down syndrome reflect amyloid deposits in the vasculature (i.e., cerebral amyloid angiopathy) that weaken the vascular endothelium and may potentiate further downstream vascular dysfunction^32^. White matter hyperintensities are associated with inflammation and white matter demyelination due to small vessel disease or disruption^33^. Infarcts reflect ischemic lesions in larger vessels; they may not be mechanistically related to amyloid and tau, but may contribute to downstream neurodegeneration and cognition^34^.

Based on previous mechanistic work, chronic exposure to soluble amyloid may impair perivascular clearance, leading to downstream amyloid deposition in the parenchyma and in the vasculature. Further, small vessel disease may be one initiator of tau phosphorylation, perhaps through an inflammatory response to damage in small vessels that upregulates kinase activity^24,28,30^. Our findings were consistent with this expected temporality. We observed the earliest inflection points at age 31 for enlarged PVS and, surprisingly, infarcts, demonstrating that these vascular abnormalities are among the earliest biomarker changes observed in Down syndrome. Amyloid deposition increased in the parenchyma (amyloid PET) and in the vasculature (microbleeds) at ages 35 and 36 years, respectively. Increased tau deposition in early Braak regions was also observed at 35 years, which is consistent with previous studies showing tau deposition in adults with Down syndrome who were amyloid-negative but accumulating amyloid over time^35^, suggesting that the emergence of amyloid and tau pathology are tightly linked together in time^36,37^. Tau deposition in middle and late Braak regions were later at 39 and 37 years, respectively. To investigate this somewhat unexpected finding, we ran a sensitivity analysis excluding one individual with a very low late Braak SUVR (< 0.5) at an older age, which may have biased the age trajectory after the inflection point lower and pushed the inflection point earlier; however, the estimated age inflection point in late Braak regions was very similar (37.5 [29.1, 45.9]). Longitudinal within-subject data in adults with Down syndrome demonstrated that middle Braak regions accumulate tau prior to late Braak regions according to the amyloid cascade^36^ White matter hyperintensities emerged at 35 years old across the brain. The temporal ordering observed is consistent with previous work in late onset AD that demonstrated that greater WMH are associated with tau burden in middle and late Braak regions, but not early Braak regions^38^. The estimated inflection point for microbleeds was not significant, similar to a previous study that did not show an increase across diagnostic groups in adults with Down syndrome^26^ and may be due to methodological limitations (e.g., GRE/SWI being more susceptible to motion artifacts than other MRI sequences, and motion artifacts being more common in older individuals with more advanced disease). Alternatively, microbleeds may start to manifest even earlier than the ages represented in these studies. Indeed, pathological studies of adults with Down syndrome suggest a particularly profound vascular amyloid profile^39^.

Regionally, parietal WMH, which may be more specific to AD compared with other lobar WMH, emerged at 35 years old, approximately 18 years prior to the average age of symptom onset in adults with Down syndrome^15^. Strikingly, in autosomal dominant AD, posteriorly distributed WMH emerged as early as 22 years before estimated onset of symptoms^22,23^. The majority of enlarged PVS were observed in the cortex (5.4 [5.0, 5.9]), while approximately one third of enlarged PVS were observed in the basal ganglia (2.6 [2.4, 2.8]), which have been associated with hypertension in adults without Down syndrome^40^. Global enlarged PVS score was strongly associated with cortical PVS (R^2^ = 0.89) and moderately associated with basal ganglia PVS (0.51), while cortical PVS and basal ganglia PVS scores were less correlated with each other (0.21). Cortical PVS emerged at age 31 [16.9, 45.0], while basal ganglia PVS emerged at age 42 [28.3, 54.9]. In relation to microbleeds, 1 participant had deep microbleed(s) in the absence of lobar microbleed(s) and 14 participants had lobar microbleed(s) in absence of deep microbleed(s). In relation to infarcts, 9 participants had deep infarct(s) in the absence of lobar infarct(s), 16 participants had lobar infarct(s) in absence of deep infarct(s), and 1 participant had both. Microbleeds outside of cortical lobes and infarcts in deep, subcortical structures were not common enough in adults with DS to reliably model regionally specific age trajectories.

Compared with men, women had greater global WMH than expected for participant age, particularly in parietal, temporal, and occipital lobes, which may be another contributor to age-specific sex differences in AD risk in adults with DS^41^. Individuals with the APOE-ε4 allele had a greater likelihood of having microbleeds than expected for age, suggesting that even in the context of amyloid precursor protein overproduction due to triplication of the 21st chromosome in Down syndrome, APOE-ε4 can still affect disease progression^42^. While hypertension was not common in study participants, it was associated with PVS scores that were greater than expected for participant age, suggesting a role of hemodynamics for clearance through the perivascular space. Potential mechanisms for the enlargement of PVS include atherosclerosis, arteriolosclerosis, and elastin dysfunction, which reduces the pliability and increases pulsatility^31^. However, autopsy studies demonstrated low prevalence of atherosclerosis and arteriosclerosis in adults with Down syndrome^39^, leaving the possibility that hypertension likely affects enlarged PVS through mechanisms related to vessel elasticity in this study. Hyperlipidemia was common but was not associated with cerebrovascular disease for a given age; hyperlipidemia may not operate as a vascular risk factor in the absence of metabolic disease (e.g., diabetes) in adults with DS. Lower BMI was associated with greater global WMH, highlighting a role of other disease related processes that affect diet, exercise, and weight^43^. While WMH may reflect Wallerian degeneration to some degree in advanced stages of late onset AD, tau burden was not associated with greater than expected WMH volume for participant age in adults with Down syndrome across the lifespan, and WMH age inflection preceded later stage tau deposition, suggesting that cerebrovascular biomarkers are upstream of advanced tau pathology. Greater age-residualized global WMH, driven by parietal, occipital, and temporal lobe WMH, was associated with a diagnosis of AD dementia. Small vessel disease in posterior brain regions is a consistent cerebrovascular process in AD pathogenesis in adults with Down syndrome^26^, adults with autosomal dominant AD^22,23^, and adults with late onset AD^20,21^. Therefore, these four cerebrovascular biomarkers may represent unique biological mechanisms, each with their own influence on disease pathogenesis and course^29,30^. Ongoing longitudinal data collection in ABC-DS will support investigations into individual-level inflection points as well as the shape and rates of these cerebrovascular disease biomarker trajectories.

This study has some limitations, including the lack of pathological validation, using temporal ordering of cross-sectional events to infer change, and the lack of correction for multiple comparisons. Autopsy studies can identify individual plaques and tangle within specific cell layers, but amyloid and tau PET scans indicate when the amount of pathology is above the limit of detection at a spatial resolution of 2 mm. However, PET imaging is the in vivo gold standard for comparison against histopathology^12^. Similarly, only a proportion of microbleeds are detected on MRI with current imaging parameter^44^ and subtle cerebral blood flow changes likely precede the formation of WMH and infarcts^45,46^. Therefore, our results may be most relevant to later manifestations of pathology that can be captured with neuroimaging. Nonetheless, we used conventional radiology tools that can inform clinical evaluation by establishing normative expectations for the measured pathologies for an individual given their age. Future work will include the identification, validation, and incorporation of biofluidic measures of vascular function^47,48^ and comparison to autopsy data that could provide more mechanistic context for the radiological markers studied here. Conclusions about temporal ordering of the emergence of radiological abnormalities were derived with methods that are similar to those used in studies of autosomal dominant AD^49,50^. In both cases, there is a nearly 100% likelihood of AD incidence in the context of overproduction and/or altered metabolism of amyloid pathology with similar variability around age of dementia onset^15^. Multiple comparison correction was not performed as our primary interest was in characterizing the natural history of markers of cerebrovascular disease among adults with Down syndrome (i.e., inflection point models). Given the relevance of these findings to therapeutic intervention strategies in adults with Down syndrome, we wanted to minimize Type 2 statistical error (i.e., false negative) to inform future mechanistic studies of any potentially relevant pathways (i.e., age-residualized models), although we recognize the possibility of inflated Type 1 error (i.e., false positive) as a limitation. Still, our findings converge with cross-sectional literature in autosomal dominant AD, showing an early and reliable increase in WMH^22,23^, enlarged PVS^51^, microbleeds^52^, and infarcts^53^, which were later confirmed longitudinally^54,55^. Further, in late onset AD, APOEε-4 was associated with microbleeds^56^, women had faster rates of deep WMH progression^57^, and enlarged PVS were dependent on arterial hemodynamics^58^ and were associated with hypertension in a spatially-dependent manner^59^. Replication studies are needed in larger, longitudinal, and external datasets. Ongoing longitudinal data collection in ABC-DS will support investigations into individual-level inflection points, which may be earlier or later compared to the group estimate, as well as the shape and rates of these cerebrovascular disease biomarker trajectories.

Furthering our understanding of AD pathogenesis with respect to cerebrovascular disease is additionally important, particularly in adults with Down syndrome, because of the common incidence of edema or hemorrhage amyloid-related imaging abnormalities (ARIA) that emerge as a results of current anti-amyloid antibody therapeutics^16^. There is evidence of cerebrovascular disease on the group level in adults with Down syndrome along the AD continuum, but some older adults have relatively low burden. Even in the absence of existing microbleeds, which are currently the only known MRI risk factor for ARIA, adults with Down syndrome may still be at increased risk because MRI only captures a proportion of the microbleeds detected at autopsy^39,52^. Future research should study the extent to which other visible cerebrovascular lesions on MRI may be used to predict who is at risk for developing ARIA. Individuals with Down syndrome may be at particular risk for these side effects in anti-amyloid therapeutics. In the absence of approved pharmacological treatments for AD in adults with Down syndrome, modifiable factors like sleep^60,61^, diet and exercise^62^, and leisure activity^63,64^ may be potential therapeutic avenues.

MRI imaging data across the lifespan demonstrate a dramatic cerebrovascular profile in adults with Down syndrome that appears to evolve developmentally in parallel with AD pathophysiology approximately two decades prior to dementia symptoms. This work joins an emerging literature that incorporates cerebrovascular disease into our understanding of AD pathogenesis and progression and highlights new avenues towards our understanding of the cause of AD, therapeutic and preventative strategies, and safety outcomes in this unique population. Future work should emphasize the potential role of cerebrovascular pathologies in AD, beyond the way by which they impact downstream neurodegeneration and cognitive impairment as simple comorbidities as they may precede and contribute to AD pathology.

## Methods

### Clinical characterization

Adults with Down syndrome from the multisite Alzheimer’s Biomarker Consortium-Down Syndrome study^65^ (ABC-DS; n = 242; age = 25–72, 45 ± 10; 43% women) underwent MRI, amyloid PET, and tau PET under the Neurodegeneration in Aging Down Syndrome (NiAD) study and the Biomarkers of Alzheimer’s Disease in Down Syndrome (ADDS) study protocols. The studies under which data were collected were approved by the institutional review boards at participating institutions (i.e., University of Pittsburgh, Columbia University Irving Medical Center, The New York State Institute for Basic Research in Developmental Disabilities/New York State Psychiatric Institute, Harvard Medical School, University of Wisconsin-Madison, University of Cambridge, University of California, Irvine), performed in accordance with the Declaration of Helsinki, and written informed consent was obtained from participants and/or their legal guardian or legally authorized representative. Every participant gave assent prior to any study-related procedure.

Clinical diagnoses were assigned by a consensus panel that included clinicians with expertise in the assessment of adults with Down syndrome^66^. One of four AD consensus diagnoses was assigned to each participant based on the results from neuropsychological testing, clinical chart reviews, and interviews with knowledgeable informants, with additional consideration of health history, functional and vocational abilities, and neuropsychiatric symptoms. Results from neuroimaging or other biomarker studies were not considered in the diagnostic formulation. A diagnosis of “cognitively-stable” (CS) indicated no evidence of clinically significant cognitive decline beyond preclinical intellectual functioning and age. A diagnosis of “mild cognitive impairment-Down syndrome” (MCI-DS) indicated evidence of cognitive decline over time beyond preclinical intellectual functioning and age, but insufficient to suggest dementia. A diagnosis of “AD dementia” indicated clear evidence of substantial cognitive and functional decline of breadth and severity greater than MCI-DS, with a high degree of confidence. Eleven participants in the neuroimaging sample (4.2%) were excluded based on complications or concerns unrelated to neurodegenerative disorders (e.g., severe sensory loss, new psychiatric diagnosis).

### Neuroimaging acquisition and analysis

Participants were scanned on 3 T MRI and PET scanners, following protocols put forth by the Alzheimer’s Disease Neuroimaging Initiative (ADNI). Participants underwent a high-resolution T1-weighted anatomical scan (repetition time [TR]/echo time [TE]/inversion time [TI] = 2,300/2.96/900 ms, voxel size = 1 × 1 × 1 mm^3^), a T2-weighted fluid-attenuated inversion recovery (FLAIR) scan (TR/TE/TI = 5,000/386/1,800 ms, voxel size = 0.4 × 0.4 × 0.9mm^3^), and a T2*-weighted gradient echo (GRE) scan (TR/TE = 650/20 ms, voxel size = 0.8 × 0.8 × 4 mm^3^) or susceptibility-weighted image (SWI; TR/TE = 27/20 ms, voxel size = 0.9 × 0.9 × 1.5 mm^3^). Participants underwent amyloid PET with [^11^C]PiB (15 mCi, 50–70 min post-injection scan, 5 min frames) or [^18^F]Florbetapir (AV45; 10 mCi, 80–100 min post-injection scan, 5 min frames); participants also underwent tau PET with [^18^F]Flortaucipir (AV1451; 10 mCi, 75–105 min post-injection, 5 min frames). All PET data were corrected for attenuation, detection dead time, scanner normalization, scatter, and radioactive decay.

Magnetic resonance imaging scans were analyzed for cerebrovascular disease with analytic pipelines developed in-house. For *white matter hyperintensities*, total and lobar (frontal, temporal, parietal, occipital) WMH volumes were semi-automatically segmented from T2-weighted FLAIR scans. Images were interpolated to a standard matrix in MNI152 space (256 × 256 × 256; 1 mm^3^), skull stripped, bias field corrected, and intensity normalized (0–255). Percentile thresholds initiated a Gaussian mixture model, separating dark/bright and bright/brightest intensities; estimated percentile thresholds were then relaxed by an intensity of 10 to account for variations in FLAIR quality. Roberts edge detection removed any hyperintense labels from non-white matter before visual inspection. Enlarged perivascular spaces (PVS) were visually inspected on T1-weighted scans and classified as hypointensities across 13 brain regions, rated from 0 to 2 based on FLAIR characteristics (hyperintense ring), and combined into a global score ranging from 0 (no enlarged PVS in any region) to 26 (most severe enlarged PVS in each region)^67,68^. We developed an algorithm^58^ based on anatomical location, appearance on FLAIR, and size to determine the most likely underlying pathology of a given lesion. A hyperintense FLAIR rim around a T1 void is by far the single most important determining factor to distinguish enlarged PVS from infarcts, although the need for a FLAIR rim is lower in areas in which enlarged PVS rarely exist (such as the brain stem or the upper basal ganglia). *Microbleeds* were visually rated as hypointense round or ovoid lesions on GRE or SWI, surrounded at least halfway by parenchyma with a “blooming” effect and no hyperintensity on accompanying T1-weighted or FLAIR scans to distinguish them from iron or calcium deposits, bone, or vessel flow voids. Due to a skewed distribution (i.e., if present, most scans had 1 microbleed), microbleeds were scored across the whole brain as present or not present. *Infarcts* were visually rated on T2-weighted FLAIR scans as discrete hypointense lesions greater than 5 mm with a partial or complete hyperintense ring, confirmed on T1 scans as hypointense areas, and scored across the whole brain as present or not present due to a skewed distribution. Amyloid PET with [^11^C]PiB or [^18^F]Florbetapir were harmonized into the centiloid scale using reported formulas available on Global Alzheimer’s Association Information Network (GAAIN; http://www.gaain.org)^69,70^. Tau PET with [^18^F]Flortaucipir was quantified as standard uptake value ratio (SUVR, 80–100 min post-injection, FreeSurfer-defined cerebellar gray matter reference region) in Braak I/II, Braak III/IV, and Braak V/VI for early, middle, and late tau burden. Biomarkers were available in 242 participants for WMH, 182 participants for enlarged PVS score, 140 participants for microbleeds, and 237 participants for infarcts; 215 participants for amyloid PET, and 175 participants for tau PET.

### Genotyping

Participants were genotyped for APOE (rs429358 and rs7412) with the Kompetitive allele-specific polymerase chain reaction genotyping system (LGC Genomics; Berlin, Germany). For these analyses, individuals with at least one copy of the APOE-ε4 allele were classified as APOE-ε4 carriers.

### Statistical analysis

Age, amyloid burden, and tau burden were compared across clinical diagnostic groups to support the use of chronological age as disease progression in adults with Down syndrome, while demographic characteristics (i.e., sex, premorbid intellectual developmental disability, APOE status) and vascular risk factors (i.e., hypertension, diabetes, hyperlipidemia, body mass index (BMI), and obstructive sleep apnea (OSA)) were compared across clinical diagnostic groups to assess potential covariates. Vascular risk factors were reported by participants or their informants as part of their health history (“Do you currently have or have you ever had a diagnosis of [disease]”) or obtained from medical/health records. Information was aggregated across sources and binarized as “yes” or “no”. Body mass index was objectively measured and used continuously. Cerebrovascular biomarkers were fit with piece-wise, left null regression models against age to estimate the age inflection point at which these cerebrovascular markers emerge, adjusting for study protocol (i.e., NiAD vs ADDS). The Davies test^71^ was used to determine if the slope after the estimated inflection point was different from the slope before the estimated inflection point (i.e., zero). General linear models were used for continuous variables (i.e., WMH, enlarged PVS) and logistic regression models were used for dichotomous variables (i.e., the presence of microbleeds, the presence of infarcts). Log transformations for skewed variables (i.e., WMH volume) and multisite harmonization methods (e.g., ComBat^72^) were explored, and led to minimal improvements in model fits with similar results; therefore, untransformed WMH volumes were used and study was included as a simple covariate in reported models. Further, the interpretation of the inflection point as the age at which cerebrovascular biomarkers visibly emerge on MRI is preserved in comparison to the age at which a transformed variable deviates from zero.

As amyloid precursor protein is overexpressed from birth in adults with Down syndrome, age can reasonably represent disease duration and cross-sectional models can provide pseudo-longitudinal trajectories of cerebrovascular biomarker development^11,15^. Amyloid chronicity^73^ has also been shown to represent disease progression beyond chronological age, but would limit our sample size to those with amyloid PET. To determine the emergence of cerebrovascular disease relative to classical AD biomarkers, including amyloid and tau, we also fit left null regression models with age for amyloid PET Centiloids and tau PET SUVRs.

Traditional vascular risk factors, including hypertension and diabetes type 2, are lower in adults with Down syndrome compared with adults without Down syndrome; however, some vascular risk factors are present at similar or higher rates, including hyperlipidemia, high BMI, and OSA. The residuals from the piecewise left-null regression against age (i.e., higher or lower biomarker level than expected for a given age) were fit against demographic data, vascular risk factors, amyloid and tau burden, and AD diagnosis (Cognitively-Stable (reference group), MCI-DS, AD dementia) to determine their influence on the development of each cerebrovascular disease biomarker. All statistics were run in R v4.2.2.

## Data Availability

ABC-DS is committed to providing rapid public access to all clinical, cognitive and biomarker (fluid and imaging) data, without embargo, and access to the biological samples by qualified scientific investigators. ABC-DS data are transferred to the Laboratory of Neuro Imaging (LONI), for harmonization, documentation and de-identification; biospecimen samples are transferred and managed by the National Centralized Repository for Alzheimer’s Disease and Related Dementias (NCRAD) and an ABC-DS biospecimen bank. As of May 2021, data from the first and second waves of longitudinal data are available for requests. Qualified investigators can submit requests for access to data and samples (https://pitt.co1.qualtrics.com/jfe/form/SV_cu0pNCZZlrdSxUN), and all requests will be reviewed by ABC-DS investigators and NIH staff. Approved data requests will be managed by the ABC-DS Biostatistics and Data Management Core for access to the clinical, cognitive, and neuroimaging data listed below. Upon approval and availability of biospecimen samples, NCRAD will distribute DNA, plasma and serum, and the ABC-DS biospecimen bank will distribute CSF. LONI will store the associated data for access by approved investigators (https://ida.loni.usc.edu/collaboration/access/appLicense.jsp;jsessionid=AC572158DA02C57FD870AE42D137FFF0).
